# Editorial: Physical activity in people with mental disorders: Benefits, risks and prescription

**DOI:** 10.3389/fpsyt.2023.1189053

**Published:** 2023-03-30

**Authors:** Huixuan Zhou, Xinfeng Tang, Weijun Zhang, Yi-lang Tang

**Affiliations:** ^1^Department of Physical Fitness and Health, School of Sport Science, Beijing Sport University, Beijing, China; ^2^Key Laboratory of Exercise and Physical Fitness, Ministry of Education, Beijing Sport University, Beijing, China; ^3^Department of Psychology, Renmin University of China, Beijing, China; ^4^School of Social Development and Public Policy, Beijing Normal University, Beijing, China; ^5^Department of Psychiatry and Behavioral Sciences, Emory University School of Medicine, Atlanta, GA, United States; ^6^Mental Health Service Line, Atlanta Veterans Administration Medical Center, Decatur, GA, United States

**Keywords:** physical activity, sports, mental disorders, depression, anxiety, cognitive impairment, exercise prescription, health promotion

Physical activity is an essential component of a healthy lifestyle and it has benefits for the prevention and treatment of various physical and mental health conditions (1). However, people with mental disorders often encounter barriers and challenges to participating in regular physical activity. Furthermore, people with the risk of mental disorders are often less properly advised on this matter, together with concerns regarding the potential risks and difficulties of uptake and adherence to physical activity (2). For instance, depressive symptoms have been found to be associated with less participation in physical activity and lower adherence to physical activity regimens recommended by physicians, owing to feelings of insufficient energy and lack of interest and motivation (3). The COVID-19 pandemic has changed the world in many fundamental ways, it has increased stress and uncertainty, and social distancing, quarantine, and lockdown measures have led to reduced physical activity. These factors have also negatively affected mental health outcomes, such as depression, anxiety, and cognitive impairment (4, 5). The relationship between physical activity and mental health during the pandemic era is of great relevance.

In this Research Topic, we investigated the benefits of physical activity for people with mental disorders, and the risks or barriers they encounter when participating in exercises; and exercise or physical activity prescriptions for preventive or therapeutic purposes of mental disorders, especially for people who are restricted in indoor living space (e.g., people in quarantine or telecommuting, people receiving medical treatment or nursery care at home due to limited healthcare resources).

There are three systematic reviews, four intervention studies, and five cross-sectional studies published on this Research Topic (see Table 1).

**Table 1:** List of articles.

**Type**	**Title**
Systematic review	Tang et al. Effects of aquatic exercise on mood and anxiety symptoms: A systematic review and meta-analysis. *Front Psychiatry*. 13:1051551.
	Wang et al. The influence of exercise interventions on cognitive functions in patients with amnestic mild cognitive impairment: A systematic review and meta-analysis. *Front Public Health*. 10:1046841.
	Hirschbeck et al. Psychiatric medication and physical performance parameters—Are there implications for treatment? *Front Psychiatry*. 13:985983.
Intervention study	Zhao et al. Personalized individual-based exercise prescriptions are effective in treating depressive symptoms of college students during the COVID-19: A randomized controlled trial in China. *Front Psychiatry*. 13:1015725.
	Li R. et al. Effect of resistance training on heart rate variability of anxious female college students. *Front Public Health*. 10:1050469.
	Lei et al. An exercise prescription for patients with lung cancer improves the quality of life, depression, and anxiety. *Front Public Health*. 10:1050471.
	Robertson et al. Acute electroencephalography responses during incremental exercise in those with mental illness. *Front Psychiatry*. 13:1049700.
Cross-sectional study	Liu Y. et al. The effect of different types of physical activity on cognitive reaction time in older adults in China. *Front Public Health*. 10:1051308.
	Li C. et al. Health benefits of physical activity for people with mental disorders: From the perspective of multidimensional subjective wellbeing. *Front Psychiatry*. 13:1050208.
	Yuan et al. The after-school sedentary behavior status among children and adolescents with intellectual disabilities. *Front Psychiatry*. 13:1049180.
	Liu Z. et al. Physical activity levels associated with insomnia and depressive symptoms in middle-aged and elderly patients with chronic schizophrenia. *Front Psychiatry*. 13:1045398.
	Chen et al. The relationship between motor development and social adaptability in autism spectrum disorder. *Front Psychiatry*. 13:1044848.

The systematic reviews and meta-analyses from Tang et al. and Wang et al. indicated that aquatic exercise played a positive role in mental health, and exercise could improve global cognitive function and several specific cognitive functions in patients with amnestic mild cognitive impairment, respectively. Different from their reviews focusing on the benefits of exercises on mental health, Hirschbeck et al. reviewed the effects of psychiatric medications on physical performance and showed that stimulants had consistent performance-enhancing effects on patients with psychiatric disorders and well-trained subjects, while other psychotropic drugs showed different effects in various studies.

Three intervention studies showed the positive effects of exercise prescriptions on mental health. Zhao et al. indicated that personalized exercise prescriptions could improve adherence to interventions and reduce serious adverse events for college students with depressive symptoms. Li R. et al. showed that the 8-week resistance training could increase the heart rate variability in anxious female college students and improve their autonomic nervous disorder, and Lei et al. showed that the 8-week exercise prescription of *Baduanjin* was an effective supportive treatment for lung cancer patients with depression and anxiety. The other intervention study from Robertson et al. aimed to understand the potential mechanism of positive effects of physical activity on depression symptoms, which showed that increased prefrontal cortex gamma during exercises could differentiate between people with and without mental disorders.

Cross-sectional studies in this Research Topic explored the correlation between physical activity or sedentary behavior and mental health outcomes. Liu Y. et al. found that older adults could delay the decline in cognitive reaction time, if they maintained a moderate level of physical activity in both leisure and work time physical activity. Li C. et al. showed that physical activity decreased the severity of depression by improving life satisfaction and making a sense of purpose and meaning in life. Yuan et al. found that children and adolescents with intellectual disabilities spent a long time on after-school sedentary behavior, which is concerning. Liu Z. et al. found that low physical activity levels may be a risk factor for comorbid insomnia and depressive symptoms in patients with chronic schizophrenia. Chen et al. found that the social adaptability of autistic children may be improved by the development of fine motor, which could be an early focus in the interventions for children with autism spectrum disorder.

In summary, studies included in this Research Topic showed a positive association between exercise and mental health outcomes, and the benefits of some exercise prescriptions on patients with mental disorders. Meanwhile, sedentary behavior or lack of physical activity may negatively impact the mental health of some populations. Physical activity is suggested to be added as a routine practice to clinical care or intervention for patients with mental disorders. Given the lack of evidence of some exercise interventions, longitudinal studies are further needed to verify the effects of various exercises. In addition to clinical trials examining the efficacy of exercises, mental health promotion programs including exercise should be conducted, and the effectiveness of exercise prescriptions in the real-world needs to be studied.

## Author contributions

HZ and XT prepared the manuscript. WZ and Y-lT revised the manuscript. All authors contributed to the article and approved the submitted version.

## References

[1] Jurak G Morrison SA Leskošek B Kovač M HadŽić V Vodičar J. Physical activity recommendations during the coronavirus disease-2019 virus outbreak. J Sport Health Sci. (2020) 9:325–7. 10.1016/j.jshs.2020.05.003 32426171 PMC7229466

[2] Marashi MY Nicholson E Ogrodnik M Fenesi B Heisz JJ. A mental health paradox: Mental health was both a motivator and barrier to physical activity during the COVID-19 pandemic. PLoS ONE. (2021) 16:e0239244. 10.1371/journal.pone.0239244 33793550 PMC8016471

[3] Schuch FB Vancampfort D. Physical activity, exercise, and mental disorders: It is time to move on. Trends Psychiatry Psychother. (2021) 43:177–84. 10.47626/2237-6089-2021-0237 33890431 PMC8638711

[4] Nochaiwong S Ruengorn C Thavorn K Hutton B Awiphan R Phosuya C. Global prevalence of mental health issues among the general population during the coronavirus disease-2019 pandemic: A systematic review and meta-analysis. Sci Rep. (2021) 11:10173. 10.1038/s41598-021-89700-8 33986414 PMC8119461

[5] Zach S Zeev A Ophir M Eilat-Adar S. Physical activity, resilience, emotions, moods, and weight control of older adults during the COVID-19 global crisis. Eur Rev Aging Phys Act. (2021) 18:5. 10.1186/s11556-021-00258-w 33648448 PMC7917372

